# Breast Reconstruction: The Oncoplastic Approach

**DOI:** 10.3390/jcm13164718

**Published:** 2024-08-12

**Authors:** Vincenzo Vindigni, Francesco Marena, Chiara Zanettin, Franco Bassetto

**Affiliations:** Unit of Plastic and Reconstructive Surgery, University of Padova, Via Giustiniani 2, 35128 Padova, Italy; vincenzo.vindigni@unipd.it (V.V.); chiara.zanettin@aopd.veneto.it (C.Z.); franco.bassetto@unipd.it (F.B.)

**Keywords:** breast reconstruction, oncoplasty, breast conservation surgery, autologous reconstruction

## Abstract

Breast reconstruction surgery is continualladvancing, significantly enhancing patient well-being. Current surgical techniques prioritize minimizing donor site morbidity while achieving a more natural breast appearance. Increasing patient preferences for avoiding prosthetic materials in reconstruction, along with advancements in oncological safety and heightened aesthetic expectations, are driving the exploration and development of innovative approaches. Today’s reconstructive options range from straightforward oncoplastic glandular remodeling to intricate microsurgical procedures. This narrative review, titled “Breast reconstruction: the oncoplastic approach,” provides a comprehensive overview of contemporary trends in breast-conserving treatment. It evaluates the indications for these techniques and offers guidance to plastic surgeons in crafting personalized treatment plans. This approach presents a valuable single-stage alternative or adjunct to traditional prosthetic or microsurgical reconstruction methods.

## 1. Introduction

### 1.1. The Development of Breast Oncologic Surgery

Breast cancer remains the most prevalent cancer among women in many countries, affecting up to one in eight females. The surgical treatment of breast cancer has undergone significant changes over the years, transitioning from radical mastectomy and axillary node dissection to more conservative treatments for the breast and axilla, as it has been observed that less radical treatments do not impact survival rates. Breast conservative treatment (BCT) has evolved to include non-oncological mammoplasty techniques, providing a variety of methods for treating breast cancer [1,2]. According to Jonczyk et al. [3], data from the National Surgical Quality Improvement Program (NSQIP) database indicates that oncoplastic reconstruction increased by 241% between 2005 and 2016, with an average annual increase of 11%. Encouraging results related to patient satisfaction, oncological safety, and cost-effectiveness [4] have led to growing approval among surgeons [5].

Initially, mastectomy was associated with delayed reconstruction using myocutaneous flaps. However, immediate breast reconstruction techniques now allow for the preservation of skin and nipples, incorporating breast prostheses. Advances such as dermal matrices, prepectoral prostheses, and fat grafts have been implemented to improve outcomes. In some centers, robotic and video-assisted mastectomies are performed [6]. These innovative techniques aim to minimize external wounds on the breast surface, typically placing the scar in the axillary fold. They also demonstrate lower rates of skin vascular issues and nipple–areola complex ischemia compared to traditional nipple-sparing mastectomies [7]. The cost-effectiveness of robotic mastectomy is currently being investigated in clinical trials, considering the extended surgical time required and its implications. Given the lack of definitive data, these procedures should be performed only by experienced surgeons in specialized robotic centers [8].

While axillary surgery has decreased, the variety of breast surgeries has expanded, necessitating comprehensive knowledge and training in the range of surgical options. The advent of neoadjuvant chemotherapy, which allows for initial tumor reduction and resection of residual disease, has increased the rate of breast-conserving therapy (BCT) [9]. The traditional focus on tumor size has shifted to a consideration of the breast-to-volume ratio. Oncoplasty represents a new paradigm that breast surgeons should adopt and master [10]. Up to 30% of patients who underwent quadrantectomy requested late reconstruction or revision due to unsatisfactory aesthetic outcomes, highlighting the importance of aesthetic considerations in the initial surgical stage [11]. Studies have shown that oncoplastic surgery does not significantly differ in patient survival rate compared to partial or complete mastectomy [12]. It has demonstrated comparable or lower rates of complications and recurrence, as well as higher patient satisfaction, compared to traditional mastectomy and reconstruction techniques [13,14].

### 1.2. Oncoplastic Breast-Conserving Surgery (OBCS)

From an oncological point of view, OBCS allows initial candidates for radical treatment to receive a more conservative one, obtaining lower rates of positive margins without compromising aesthetic results [15].

Breast-conserving therapy (BCT), or BCS followed by radiation therapy, has demonstrated equivalent survival outcomes compared to mastectomy in randomized prospective trials [16,17]. The aesthetic issues of the conserving approach, however, are not always insignificant [18], especially when adjuvant radiation therapy is needed.

OBCS has achieved good cosmetic results even in more extensive resections of locally advanced carcinomas, representing a satisfactory option to avoid radical surgery, whose morbidity is higher [19]. Any patient eligible for breast-conserving surgery, with appropriate size and ptosis in relation to tumor size, should be considered a candidate for OBCS [20,21].

## 2. Oncoplastic Strategies and Classification

Oncoplastic breast-conserving surgery (OBCS) integrates plastic surgery principles into breast cancer treatment, ensuring that the tumor excision is planned alongside breast reshaping to prevent future local deformities. Additionally, by reducing the parenchyma, oncoplastic techniques enhance the effectiveness of radiotherapy in the remaining tissue, ensuring dose homogeneity and low complication rates [22,23,24].

Andrade Urban [9] proposed a classification system based on the surgical skills required:

Class I involves glandular mobilization and reshaping, which does not require specialized surgical training.

Class II requires specific training for procedures involving breast reconstruction with implants, mastoplasty, and bilateral mastopexy for symmetrization.

Class III encompasses the use of autologous flaps or a combination of techniques, necessitating specific reconstructive training.

Other classifications for oncoplastic procedures have also been suggested. 

Clough et al. categorize the techniques into two levels based on the tumor-to-breast volume ratio and the complexity of the reconstruction [20,25]:

Level 1 involves glandular mobilization and repositioning of the nipple–areola complex, with less than 20% of the breast volume resected.

Level 2 involves resections ranging from 20% to 50% of the breast volume, including volume repositioning techniques (therapeutic mammoplasty) and volume replacement techniques (fascia or myocutaneous flaps), potentially combined with contralateral mammoplasty.

The American Society of Breast Surgeons [26] has adopted this classification for practical guidance in preoperative planning. The Clough classification was endorsed as the standard for clinical practice at the “First International Consensus Conference on Standardization of Oncoplastic Breast Conserving Surgery” [27]. However, while experts supported the classification by Hoffmann et al. [28] for billing purposes, they did not agree on a single classification for clinical research [27].

Weber et al. [29] introduced nomenclature and algorithms to standardize OBCS procedures. These include the following:

Conventional tumorectomy: Glandular reapproximation and direct wound closure.

Mastopexy: Non-oncological skin resection and nipple repositioning, with or without pedicles.

Oncoplastic tumorectomy: Glandular reshaping and volume replacement.

Oncoplastic reduction mammoplasty: Non-oncological breast reduction with nipple–areola complex repositioning via pedicles.

The proposed algorithms focus on surgical planning based on breast size and shape, tumor size and location, and vascular supply to suggest flaps, glandular reshaping, and specific pedicles for volume replacement.

The term OBCS encompasses a range of surgical procedures with varying levels of complexity. The association of oncological safety with cosmetic outcomes is relatively recent and requires further improvement [21,30]. Breast asymmetry following breast-conserving surgery is common and has been associated with poorer postoperative quality of life and psychosocial functioning. Therefore, cosmetic outcomes have become a crucial consideration in the surgical treatment of breast cancer [31].

To maintain symmetry, many patients undergo oncological treatment involving OBCS and contralateral symmetrization in the same procedure; however, the literature on what should be the right time for contralateral surgery is still debated, making it difficult to draw any conclusions about the true impact on women. The ideal timing for contralateral symmetrization surgery, given the side effects of radiotherapy (such as fibrosis and skin shrinking), should be after the end of the whole oncological treatment [32]. Waiting for a second operation and a prolonged period of asymmetry of one’s breasts can cause discomfort for patients, which often outweighs maximizing the aesthetic result. 

Oncoplastic techniques adhere to the aesthetic breast surgery principles in terms of incision location, breast shape, volume, inframammary fold (IMF) and nipple position, upper- and lower-pole fill ratios, and symmetry. When possible, oncologic access is achieved through aesthetic breast incisions. These include variations of vertical, periareolar, and inverted-T incisions.

Traditional incisions cannot be used to treat upper-pole tumors requiring skin excision; instead, other methods such as batwing mastopexy [33] or volume replacement procedures can be used.

Second, to minimize contour deformities that would otherwise be accentuated by radiation, an aesthetic breast shape can be achieved through tissue rearrangement or volume replacement to close dead space.

In a prospective study, Veiga et al. found that when comparing aesthetic results in standard BCS versus oncoplastic surgery, shape was rated the lowest [34].

While preserving symmetry, breast volume considerations are tailored as much as possible to the patient’s desired size. According to studies, you should aim for a slightly larger breast (about 10–20%) on the therapeutic side to accommodate for the volume loss that results from radiation fibrosis [35]. Additionally, radiation can cause IMF asymmetry and nipple–areolar complex (NAC) retraction, emphasizing the importance of obliterating dead space to reduce fibrosis and scar contracture.

### 2.1. Preoperative Considerations on Oncoplastic Breast Surgery [32]

Unsatisfactory cosmetic outcomes following breast conservation surgery have been associated with several factors, including patient age over 60 years; tumors classified as T2 or more advanced; the necessity for resecting more than 100 cm^3^ of breast tissue; small breast size; pronounced ptosis; prior inadequate surgical margins; improper scar orientation; tumor location in the central, medial, or lower quadrants; and radiation dose inhomogeneity [36,37,38,39,40]. The literature indicates that lumpectomy without appropriate reshaping is more likely to result in maldistribution of breast tissue and distortion of the nipple–areola complex (NAC), particularly due to scarring and post-radiation fibrosis. The oncoplastic approach can address these limitations by maintaining breast symmetry while reducing its volume. Consequently, the default reconstructive goal should extend beyond merely preserving the patient’s current appearance.

The objective of this surgery is to minimize the number of procedures, donor site morbidity, hospital admission time, complications, and failure rates, while achieving a satisfactory cosmetic outcome. The selection of the reconstructive technique should always involve a careful evaluation of the risk-to-benefit ratio. Even small tumor resections can lead to significant breast deformity, particularly when involving the lower quadrants. Post-operative scarring and retraction may result in the classic bird’s beak deformity, characterized by lower-pole shrinkage and inferior displacement of the NAC.

### 2.2. Timing of Surgery [32]

Likewise for any reconstructive technique, different timings of oncoplastic breast surgery have been proposed [41,42]:

Immediate: Oncoplastic reconstruction follows the demolition in the same definitive OBS procedure at the time of tumor resection. Revision surgery may be needed in the case of unclear definitive margins.

Delayed–immediate: OBS is delayed until pathologic margins are confirmed to be clear. The procedure usually takes place within a month of the prior surgery, and it precedes radiotherapy.

Delayed: No reconstructive procedure is performed until completing any adjuvant therapy, usually 1–2 years later. This approach guarantees no delay for oncological treatment, but it is burdened by the highest complication rates and least favorable aesthetic outcome.

The ideal timing for contralateral breast surgery would be after the eventual adjuvant radiotherapy is performed. Irradiation affects the index breast with variable degree of fibrosis, loss of volume and elasticity, so delaying the reconstructive procedure should guarantee a stabilized model for symmetrization. Nevertheless, better symmetry can be achieved in this staged approach, but few patients choose this path. A single-stage procedure represents a less impactful option.

Patients are in any case offered further refinements in a second delayed procedure if requested.

## 3. Surgical Techniques

The choice of oncoplastic surgery technique depends on multiple factors, including tumor size and location, breast size, and patient preferences.

Oncoplastic procedures are broadly categorized into volume displacement and volume replacement techniques.

Volume displacement procedures involve the glandular redistribution of the residual breast parenchyma post-breast-conserving-surgery (BCS) to restore breast contour and shape through aesthetically placed incisions. Level 1 reconstructive procedures are typically performed when less than 20% of the breast volume is excised, whereas Level 2 procedures are indicated when 20–50% of the breast volume is removed [25,26].

Level 1 techniques include limited glandular undermining and dead-space closure, suitable for patients with smaller breasts or those who prefer to avoid additional scarring. Level 2 procedures often incorporate mastopexy or reduction mammoplasty incisions and dermoglandular pedicles to preserve the nipple–areola complex (NAC) and reshape the breast mound. These patients usually have medium-to-large breasts with some degree of ptosis and sufficient glandular tissue remaining after BCS.

Volume displacement techniques typically result in a reduced overall breast size, and this reduction should be discussed with the patient beforehand.

Volume replacement techniques use regional or remote tissue to restore volume, contour, and/or skin in patients with small-to-medium-sized breasts and inadequate residual glandular tissue after BCS for volume displacement. These procedures are most often indicated for oncoplastic surgery when more than 50% of the breast tissue is removed [26].

However, volume replacement techniques can also be useful for smaller resections (20–50%) using aesthetic breast incisions such as periareolar, circumvertical, and Wise/inverted-T patterns of breast tissue in patients with smaller breasts who wish to preserve their breast size. Volume asymmetry can be difficult to conceal, and restoring contour can be challenging with glandular rearrangement, even with smaller resections in smaller breasts.

Pedicled chest wall perforator-based flaps [27,43] are the most frequently used techniques for volume replacement. Depending on the defect location, a variety of flaps based on the thoracodorsal, lateral thoracic, and intercostal systems can be utilized to restore contour and volume with aesthetically favorable donor sites. In cases where breast-conserving surgery is desired and regional tissue is insufficient, free tissue transfer for volume replacement can also be used [44]. Small flaps based on the superficial abdominal vasculature are particularly useful in thin, small-breasted patients undergoing BCS with larger tumor-to-breast ratios who wish to preserve breast volume.

Recently, the combination of oncoplastic techniques and device reconstruction, termed the “biplanar technique,” has been described. This involves placing an implant or tissue expander after tumorectomy and glandular tissue rearrangement in a properly designed submuscular pocket. This approach broadens the indications for oncoplasty, particularly volume displacement surgery, in patients with small breasts who would otherwise not be candidates [45].

When using prosthetic devices, similar to traditional breast reconstruction, surgeons must choose between a definitive implant and a temporary tissue expander based on the patient’s characteristics and preferences. Although patients may be hesitant about a two-stage procedure, the use of tissue expanders allows for revisions and corrections of imperfections or management of complications following adjuvant radiation therapy.

**Figure 1:**
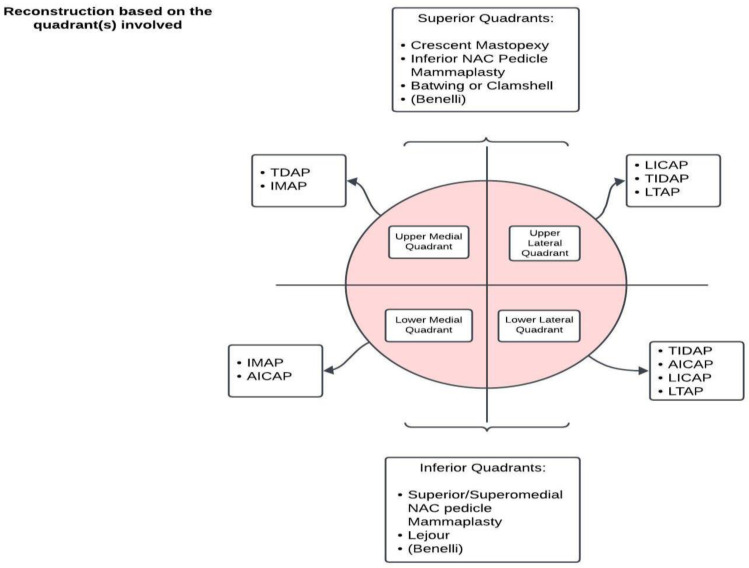
Recommendation for oncoplastic reconstruction based on the quadrant(s) involved. Scheme representing our proposal of a reconstructive algorithm focused on the disease location after a literature review and our experience [46,47].

In Figure 1 we propose an algorithm for reconstruction based on tumor location.

### 3.1. Volume-Displacing Oncoplastic Techniques 

#### 3.1.1. Glandular Tissue Rearrangement

In Level 1 procedures (<20% of breast volume excision) with non-ptotic and small-to-medium-sized breasts [26], reconstruction may be executed by separating the remaining breast parenchyma from the overlying adipose–cutaneous tissue. This allows the glandular tissue to be freed from the skin and the muscular layer to be reapproximated to close the defect. The exceeding skin has to be removed or redraped. This technique has been described as a “breastflap advancement” [46] or “breast remodelling” [48] procedure.

Attention must be paid to prevent an NAC-carrying pedicle from undermining. The glandular flaps are then mobilized and approximated with absorbable sutures to close the defect. This approach generally offers a satisfactory skin redraping and a good mound restoration with minor noticeable volume asymmetry. Preoperatively, patients should be made aware of the potential for size discrepancy, asymmetry, and residual contour deformities. A combined oncoplastic approach can be adopted in which a small submuscular implant is used to correct volume discrepancy [49].

#### 3.1.2. Oncoplastic Mastopexy and Reduction Mammoplasty

Oncoplastic reduction and mastopexy are particularly suitable for patients with moderate-to-large breasts and some degree of skin laxity. These techniques employ various skin excision patterns for tumor access while simultaneously remodeling the residual glandular tissue to restore contour and symmetry. Preoperatively, the surgeon can adapt the skin excision pattern (such as doughnut, circumvertical, or Wise pattern) and the dermoglandular pedicle for NAC repositioning, typically medial, superior, superomedial, or inferior, depending on the tumor’s location [50]. A variety of techniques, including extended and secondary pedicles, are described to address the parenchymal defect of the breast. Losken et al. have demonstrated the safety of these “autoaugmentation” techniques, reporting similar complication rates compared to standard oncoplastic procedures [51]. 

The Wise pattern mammaplasty is likely the most versatile technique for oncoplasty [52]. It allows the selection of any pedicle for the NAC and facilitates breast reshaping with multiple secondary pedicles. This design provides wide exposure of the breast, making it suitable for high-grade ptosis and significant breast volume reduction. Savalia et al. described an advanced version of the Wise pattern, termed Split reduction, which facilitates safer oncological excision and removes the same amount of skin as the traditional Wise pattern, but at the expense of a more visible scar over the tumor location instead of the inframammary fold (IMF) [32].

**Figure 2:**
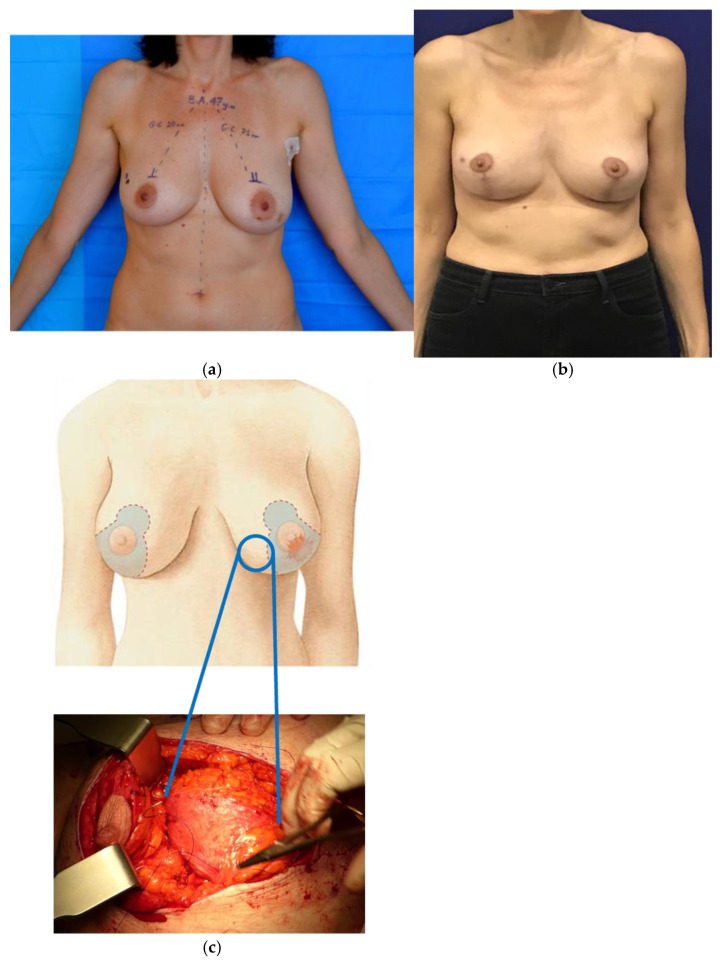
Oncoplastic reduction mammoplasty. (**a**) Preoperative assessment and measurements; (**b**) four-month postoperative evaluation; (**c**) intraoperative lumpectomy from the mastoplasty incision with preservation of the superior pedicle. Property of the Plastic Surgery Unit, Azienda Ospedaliera University of Padova, reproduced with permission.

Oncoplastic mastopexy and reduction techniques are particularly indicated for tumors located in the lower quadrants of the breast (see Figure 2) [50]. These procedures allow the lumpectomy to be performed through the skin excision pattern while preserving the NAC-carrying pedicle, especially with common superior or superomedial pedicles [53]. The vertical scar mammaplasty, as described by Lejour, is recommended for inferior central disease, lifting the NAC without any scars in the IMF. Minimal to no liposuction is suggested in oncoplastic settings to avoid potential tumor seeding [54].

Crescent mastopexy is the preferred technique for tumors of the upper pole in moderately ptotic breasts. The NAC elevation should be limited to 2 cm, marking the crescent above the areola border accordingly. The area within the crescent is excised to access the gland for resection and advancement of the glandular flaps [50]. Upper-pole tumors without skin excision may also be approached through Wise-pattern incisions, preserving the NAC on medial inferior pedicles. For upper lateral disease, an extension of the superomedial pedicle [55] or an additional inferior pedicle is used to fill the lumpectomy dead space. Similarly, for upper medial tumors, the NAC can be preserved on an inferior or medial pedicle, with the lumpectomy defect reconstructed by extending the inferior pedicle or translating medial tissue within the Wise pattern superiorly [56]. 

The batwing technique, a crescent mastopexy with two additional wings, allows extensive mastopexy without requiring pedicles for the NAC. This method is suitable for upper-pole tumors involving wide tissue areas or previously irradiated breasts where minimal tissue undermining is crucial to avoid necrosis. The clamshell technique, combining two specular batwings with the NAC centrally positioned, can be used in such cases to achieve a larger excision while using de-epithelialized tissue to replace the volumetric defect [33].

Central tumors pose challenges due to the potential involvement or resection of the NAC. For smaller periareolar tumors, a doughnut mastopexy provides access and corrects Grade I ptosis [57]. If the NAC must be removed, an inverted-T closure can be performed using a standard breast amputation technique or an inferior pedicle to provide central volume, with or without a skin paddle [58]. When skin excision is needed outside the traditional Wise pattern, incisions may need to be repositioned to more visible locations. For instance, lateral breast skin excision outside the Wise pattern can be addressed by dividing the lateral vertical limb to relocate the IMF incision onto the breast.

When the NAC is involved by the tumor, a central excision of breast tissue may be incorporated into an inverted-T mammaplasty, allowing for reshaping and immediate NAC reconstruction. This technique leverages breast ptosis to advance an inferiorly based tissue island into the central defect. It is also feasible to reconstruct an NAC on this tissue island, which can later be tattooed for completion. Alternatively, the Grisotti technique can be utilized for smaller defects, relying on the rotational advancement of a laterally based island flap with minimal reshaping of the remaining breast [59].

The circumareolar mastopexy incision, named after Louis Benelli [60], provides complete access to the breast while leaving a limited circumareolar scar, ideal for breasts with mild ptosis. The eccentric design of the outer circle lifts the NAC, vascularized by a central pedicle. This method allows a pie-shaped wedge of tissue to be easily resected from any breast location, with the defect closed with minimal undermining from the chest wall. The skin is then redraped and the incision closed with a purse-string closure around the areola.

All the techniques described above, which are the most commonly adopted in our review, should be tailored to the patient’s needs. While preoperative planning remains crucial, intraoperative flexibility in modifying the NAC-carrying pedicle or excision design is essential to address unexpected conditions following tumor removal.

### 3.2. Volume Replacement Oncoplastic Techniques

#### 3.2.1. Regional Tissue Transfer [47]

Volume replacement techniques commonly utilize pedicled flaps to restore lumpectomy defects, provide necessary skin coverage, and eliminate the need for microsurgery [61]. The advent of perforator flaps has expanded potential donor sites according to defect location while minimizing morbidity [43].

**Figure 3:**
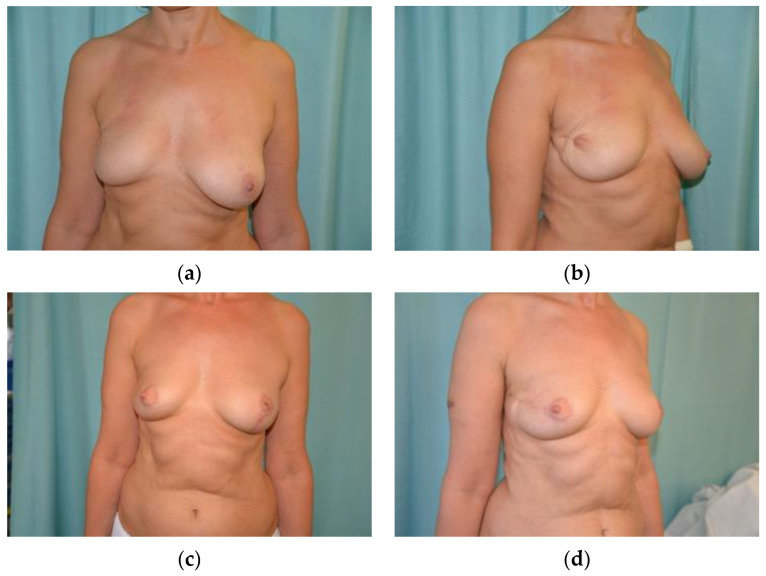
Delayed buried LICAP flap after right quadrantectomy with implant followed by radiotherapy. Contralateral mastopexy was performed. Preoperative assessment from front (**a**) and lateral view (**b**). Six months postoperative (**c**,**d**). Property of the Plastic Surgery Unit, Azienda Ospedaliera University of Padova, reproduced with permission.

The intercostal perforators offer tissue near the inferolateral breast borders, which can be isolated and mobilized into the breast footprint. The lateral intercostal artery perforator (LICAP) flap is based on perforators from the posterior intercostal arteries, typically found in the fifth to eighth intercostal spaces, approximately 3 cm anterior to the latissimus dorsi [62,63,64]. This flap can be designed transversely along the bra line or as an S-shape on the lateral thoracic roll, identified using a Doppler ultrasound. Once isolated, the flap is rotated into lateral breast defects (see Figure 3). Although the LICAP dissection is relatively straightforward, careful attention is required to avoid pneumothorax. It is particularly suitable for post-weight-loss patients and offers a skin paddle as large as 14 cm by 35 cm [65,66].

The lateral thoracic artery, the third branch of the axillary artery, provides perforators that can be isolated in the third and fourth rib spaces and harvested alone or with lateral intercostal perforators. It supplies the lateral thorax and breast, including the serratus anterior and pectoralis major muscles, and gives off a direct cutaneous branch along the lateral chest wall. The lateral thoracic artery perforator (LTAP) flaps can be identified within 2 cm of the lateral breast crease in the third and fourth intercostal spaces, above the LICAP territory [67]. The LTAP flap is versatile, allowing partial or full mobilization with minimal donor site morbidity and enabling conversion to a musculocutaneous latissimus dorsi (LD) flap intraoperatively.

The thoracodorsal artery perforator (TDAP) flap is the most described perforator flap in breast oncoplasty. It can be used with tissue expanders or implants for breast reconstruction following total mastectomy or for reconstructing partial defects after breast-conserving surgery (BCS) [68,69]. The TDAP flap allows recruitment of larger lateral chest wall tissues compared to lateral intercostal or thoracic perforators [43]. The thoracodorsal artery, branching from the subscapular artery and running alongside the thoracodorsal nerve, serves as the primary pedicle for the TDAP flap. The septocutaneous perforator is most frequently harvested for the TDAP flap [70,71,72]. Perforators from the descending branch are preferred over those from the transverse branch, and the flap is also used in immediate implant-based reconstruction [68]. TDAP flaps are suitable for lateral, inferior, and upper-lateral quadrant breast defects.

The anterior intercostal arteries branch directly from the internal mammary artery in the first six intercostal spaces, with perforators located 1 to 3 cm lateral to the sternum, moving laterally on the chest wall [62,73]. The anterior intercostal perforator (AICAP) flap supplies tissue for lower-quadrant defects. Designing the flap below the inframammary fold (IMF) enables scar concealment along the fold. The AICAP flap is recommended for outer-quadrant defects in patients with small-to-moderate-sized breasts [73].

The internal mammary artery perforator (IMAP) flap is ideal for volume replacement in the medial half of the breast when thin coverage is required [74]. Originating from the subclavian artery and running alongside the sternum, the internal mammary artery divides into the terminal musculophrenic and superior epigastric arteries [75]. Each of the first six intercostal spaces has a perforating artery accompanied by a vein and a nerve, with the second intercostal space perforator usually being dominant. The IMAP flap is versatile and provides a reliable cosmetic outcome in immediate reconstruction. However, secondary lipofilling for volume replacement and minor donor site revisions may be necessary [75,76].

#### 3.2.2. Free Tissue Transfer

**Figure 4:**
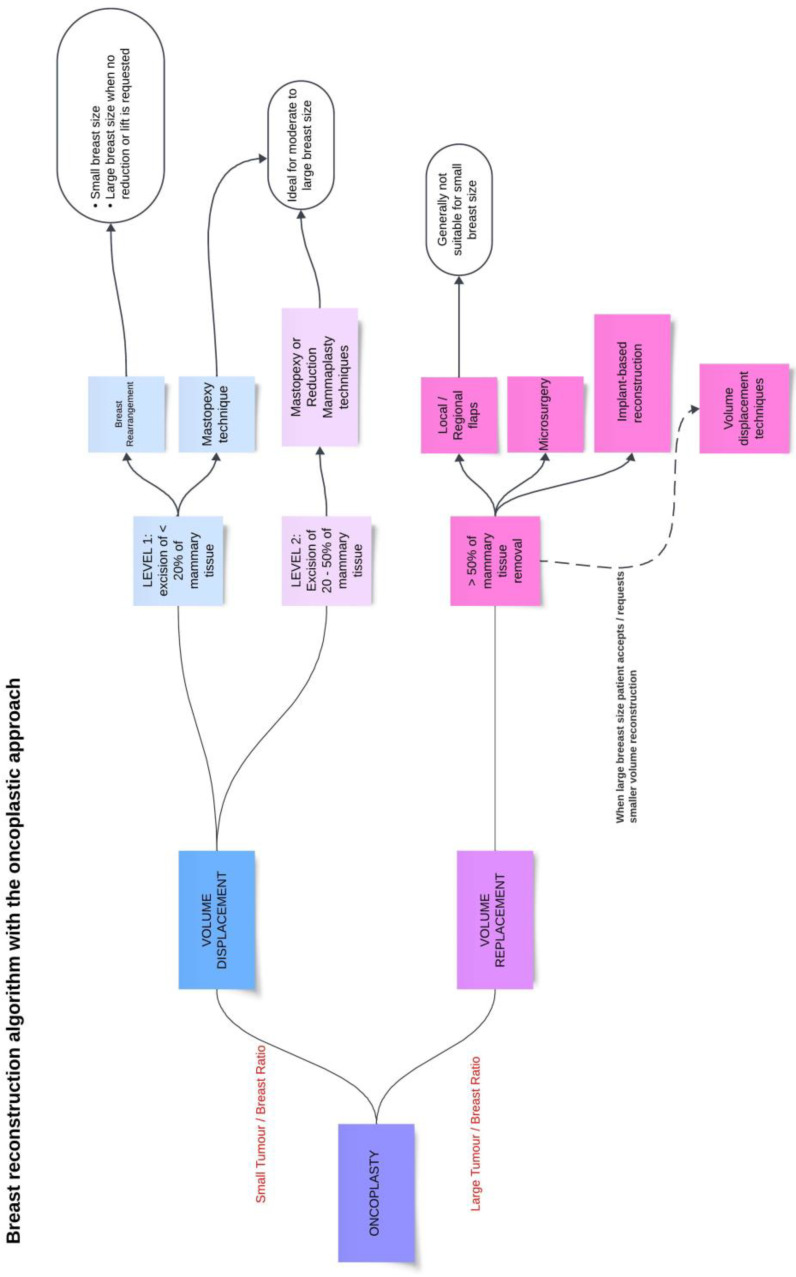
Breast reconstruction algorithm with the oncoplastic approach. Charts presenting a practical guidance for oncoplastic techniques according to the tumor/breast ratio and their indications based on the literature [2,26,46].

The planning of modern breast reconstructive surgery cannot overlook the analysis of volumes and the tumor-to-breast ratio. In Figure 4, we present a comprehensive diagram outlining the possibilities of oncoplastic surgery and its various indications. In patients with small-sized breasts and a high tumor-to-breast ratio, or those who do not accept additional chest wall scars, regional tissues may be inadequate for reconstruction. Rizzuto was the first to describe microvascular partial breast reconstruction using the superficial inferior epigastric artery (SIEA) flap [77]. Since then, various authors have reported the use of both abdominal and non-abdominal flaps for oncoplastic breast reconstruction at different time points [78,79,80].

Microsurgery offers distinct advantages in volume replacement reconstruction, including flexibility in flap design, volume and skin provision, preservation of mammary borders, and avoidance of chest wall and back scars. Particular attention must be given to incision planning, recipient vessel access, and flap selection to preserve options for future autologous breast reconstruction. Recipient vessel access is likely the most critical consideration in microvascular oncoplastic reconstruction. Limited incisions, partial defects, and restricted pedicle length depending on flap choice can present significant challenges for free tissue transfer, necessitating careful planning of incisions and flap design [81].

In the medial chest, internal mammary vessel perforators serve as excellent recipient vessels when preserved during partial mastectomy. While the main internal mammary vessels should ideally be preserved for potential future total autologous reconstruction [82], their use for partial reconstruction still permits proximal/distal approaches beyond intact branches, anastomosis to the previous pedicle, or lateral chest wall vessels, if necessary [46]. The lateral chest wall has an abundance of vessels that can be used as recipients, including intercostal perforators, the thoracodorsal serratus branch, and lateral thoracic vessels. The thoracodorsal vessels are typically avoided, when possible, to preserve the latissimus flap as a potential backup option in the future.

Multiple types of free flaps have been described for partial breast reconstruction. Traditional abdominal and thigh-based flaps, such as the deep inferior epigastric artery perforator (DIEP), Gracilis, and profunda artery perforator flaps [46], as well as the omental flap [83], have been utilized. It is advisable, when possible, to prefer the superficial system of the lower abdominal wall, including either the SIEA or the superficial circumflex iliac artery, for immediate oncoplastic reconstruction to preserve the DIEA perforators for future use if needed.

The SIEA and DIEP flap designs by Spiegel et al. [80] almost certainly preclude future abdominally based breast reconstruction. Therefore, the authors recommend against this procedure in higher-risk patients [81].

## 4. Fat Injections to the Breast: Lipomodeling

Lipomodeling or autologous fat grafting (AFG) has been widely reported as a useful additional tool in reconstructive breast surgery [84] since it guarantees a relevant improvement in cosmetic outcome after implant or autologous flap reconstruction [85]. Rigotti et al. [86] have also demonstrated that adipose-derived stem cells play a role in improving the quality of the skin over the transplanted fat, especially in irradiated fields.

The indications of AFG in reconstructive breast surgery are small-to-moderate volume augmentation, correction of localized deformities following either implant-based reconstruction or breast-conserving therapy, and complete reconstruction of the small breast with autologous fat.

Fat grafting is a simple procedure that can be carried out under general or local anesthesia. Contraindications include cases of large glandular defects or severe breast asymmetries.

The main limitations of this technique have always been the need to restrict fat volume to ease its ingrafting and the variable degree of resorption once it has been injected. Recent advancements in fat purification technology, by advanced centrifugation and filtration, which provide high-grade viability and mesenchymal fat stem cells enrichment, increase the success rate of lipofilling. In fact, mesenchymal fat stem cells enhance tissue regeneration, improving the quality and longevity of the results [87].

Several authors have proposed both immediate and delayed lipofilling in BCS as volume replacement or refinement for correcting defects or asymmetries [87,88,89,90]. Restoration of defects after BCS, especially in small-sized breasts and/or in the upper quadrants may be extremely challenging and can require volume replacement techniques [91].

After AFG was proved to be safe and reliable in breast cancer surgery, immediate adipose transfer was performed in BCS [87]. Thus, immediate lipofilling for volume replacement alone or combined with other procedures is a promising option for oncoplasty [90]. When compared with BCS alone, it has shown similar locoregional recurrence rate and fat resorption rate but higher patient satisfaction rates [92].

## 5. NAC Reconstruction

The reconstruction of the nipple–areola complex represents the final step of the breast reconstruction and should be carried out only when an acceptable symmetry and shape of the reconstructed breast has been achieved [93].

Nipple reconstructive techniques are relatively simple and can be carried out under local anesthesia.

Protuberance of the nipple can be created with local skin flaps, and once healed, color matching of both nipple and areola can be achieved by tattooing. Alternatively, a skin graft can be taken from the inner thigh using an on-lay technique [94].

The progressive flattening and loss of bulk of the reconstructed nipple have caused the abandonment of some of the nipple reconstruction techniques that had been employed in the past. Gradually, the atrophy of the flap can lead to nipple asymmetry, or worse, the bulge can almost completely disappear. For this reason, some surgeons reconstruct an over-sized and over-projected nipple to compensate for a degree of atrophy and to achieve long-term symmetry [95]. 

Currently the preferred methods of nipple reconstruction are those which use local flaps such as the skate flap or star flap. An alternative method involves a nipple prosthesis made of silicone or other materials [96].

Tattooing of the NAC can be used either as an independent technique for areola reconstruction or as a supplementary technique to achieve the best color match and correct discrepancies in size, shape, or position of the reconstructed NAC.

The advantage of the tattoo is the simplicity of the procedure, which does not require hospitalization or general anesthesia, with a very low risk of allergic and photosensitive reactions or local infections [93].

Disadvantages of the tattoo include its tendency to fade with time, frequently requiring secondary touch-ups. Proper tattooing requires training and experience to obtain optimal results [96]. 

## 6. Conclusions

Oncoplasty represents the integration of plastic surgery with oncological breast surgery principles, combining excision and reconstruction into a cohesive surgical approach. Immediate oncoplastic breast reconstruction provides optimal oncological and aesthetic outcomes in a safe, single-stage procedure, suitable for a broad range of patients.

The guiding philosophy should prioritize the least invasive approach and the most aesthetically pleasing reconstructive solution. This approach allows patients to undergo procedures with lower morbidity compared to traditional mastectomy followed by complete microsurgical reconstruction, reducing hospital stays and contributing to healthcare savings.

Current discussions highlight the trend of using implants in primary breast reconstruction. Oncoplasty offers an autologous alternative, though the simultaneous use of implants is not uncommon, with a significant emphasis on cosmetic outcomes. Volume displacement techniques are expected to be widely adopted in breast units due to their technical simplicity, flexibility, and cost-effectiveness. Older patients, where breast ptosis is more common, and patients with large breasts are ideal candidates for oncoplastic mastoplasty. The main limitation of this approach is proper patient selection: volume displacement surgery is most suitable for moderate-to-large breasts with a certain degree of ptosis, where sufficient tissue is available for reshaping. In thin patients with small or very small breasts, these techniques often result in inadequate outcomes or undesirable aesthetics.

Assessing defect location is crucial for determining when a pedicled flap is necessary. Lateral mammary defects can generally be restored with LICAP and LTAP flaps, AICAP flaps are suitable for central and lower medial defects, and IMAP flaps are better for medial and central deficiencies, even larger ones. Free tissue transfer allows for the preservation of breast volume in women with limited regional resources, broadening the indications to patients who might not otherwise be good candidates for oncoplasty.

Attention must be paid to incisions, flap design, and balancing a good pedicle with preserving potential donor sites for future total breast reconstruction. Further studies will refine technical details and indications to better meet patient expectations.

Considering all patient and tumor characteristics is critical. We emphasize the importance of a multidisciplinary team, including breast surgeons, plastic surgeons, medical oncologists, radiologists, pathologists, radiotherapists, and genetic counselors. A multidisciplinary approach, with careful assessment of the neoplasm and collegial discussion of the therapeutic strategy, ensures successful breast-conserving surgery. Patients appreciate having a lead referral figure, usually the breast surgeon in our unit, who manages their care throughout the entire therapeutic pathway.

Additionally, volume displacement techniques and contralateral symmetrization procedures offer the opportunity to treat concomitant gigantomastia or ptosis, improving the overall appearance of the breasts. It is essential to consider surgical alternatives to address intraoperative modifications. Volume replacement techniques with regional pedicled flaps can restore a pleasant breast contour when a wider excision is needed and can be implemented in all surgical settings, even without microsurgery.

In prosthetic reconstruction, lipofilling is routinely offered and performed for many patients in the oncoplastic setting. We have observed excellent results in terms of symmetry with fat injections as a refining secondary procedure, especially after volume replacement techniques. However, some women are reluctant to undergo additional surgery intended as an extension of the oncological pathway. Despite the general surgical risks associated with delayed procedures, we are confident in the safety and efficacy of lipofilling in oncological reconstruction. However, new techniques of transferring adipose tissue enriched with stem cells still lack long-term studies to confirm their safety definitively.

## Data Availability

No new data were created. All information available on PubMed.
